# High Prevalence of Asymptomatic Neurocysticercosis in an Endemic Rural Community in Peru

**DOI:** 10.1371/journal.pntd.0005130

**Published:** 2016-12-19

**Authors:** Luz M. Moyano, Seth E. O’Neal, Viterbo Ayvar, Guillermo Gonzalvez, Ricardo Gamboa, Percy Vilchez, Silvia Rodriguez, Joe Reistetter, Victor C. W. Tsang, Robert H. Gilman, Armando E. Gonzalez, Hector H. Garcia, The Cysticercosis Working Group in Peru

**Affiliations:** 1 Cysticercosis Elimination Program and Center for Global Heath - Tumbes, Universidad Peruana Cayetano Heredia, Tumbes, Peru; 2 UMR 1094 Inserm Neuroépidémiologie Tropicale (NET), Institut d'Epidémiologie et de Neurologie Tropicale (IENT), Faculté de Médecine de l'Université de Limoges, Limoges, France; 3 Epidemiology Unit, Hospital Regional II-2 Tumbes, Tumbes, Peru; 4 Department of Public Health and Preventive Medicine, Oregon Health & Science University, Portland, Oregon, United States of America; 5 Instituto de Ciencias Neurológicas, Lima, Peru; 6 Georgia State University, Atlanta, Georgia, United States of America; 7 Department of International Health, Bloomberg School of Public Health, Johns Hopkins University, Baltimore, Maryland, United States of America; 8 Department of Microbiology, School of Sciences, Universidad Peruana Cayetano Heredia, Lima, Peru; Instituto de Investigaciones Biomedicas, UNAM /Instituto de Neurologia y Neurocirugía, MEXICO

## Abstract

**Background:**

Neurocysticercosis is a common helminthic infection of the central nervous system and an important cause of adult-onset epilepsy in endemic countries. However, few studies have examined associations between neurologic symptoms, serology and radiographic findings on a community-level.

**Methodology:**

We conducted a population-based study of resident’s ≥2 years old in a highly endemic village in Peru (pop. 454). We applied a 14 -question neurologic screening tool and evaluated serum for antibodies against *Taenia solium* cysticercosis using enzyme-linked immunoelectrotransfer blot (LLGP-EITB). We invited all residents ≥18 years old to have non-contrast computerized tomography (CT) of the head.

**Principal findings:**

Of the 385 residents who provided serum samples, 142 (36.9%) were seropositive. Of the 256 residents who underwent CT scan, 48 (18.8%) had brain calcifications consistent with NCC; 8/48 (17.0%) reported a history of headache and/or seizures. Exposure to *T*. *solium* is very common in this endemic community where 1 out of 5 residents had brain calcifications. However, the vast majority of people with calcifications were asymptomatic.

**Conclusion:**

This study reports a high prevalence of NCC infection in an endemic community in Peru and confirms that a large proportion of apparently asymptomatic residents have brain calcifications that could provoke seizures in the future.

## Author Summary

Neurocysticercosis is a parasitic infection of the brain and a common cause of epilepsy in many countries in Latin America, Asia and Africa. In this study, we applied a combination of head CT, serology and symptoms screening in a rural village in northern Peru. We found that the infection was very common in this community, where nearly one in five adults had calcified lesions in the brain. While the majority of these people reported never had experienced seizures, brain calcifications are known to result in chronic intermittent seizures in some. Follow-up studies are needed to help understand how many people with calcifications will go on to experience seizures in their lifetime, and to better predict those who are at greatest risk so that preventive intervention can be offered.

## Introduction

Neurocysticercosis (NCC) is a common helminthic infection of the central nervous system and the cause of late-onset epilepsy in many lower and middle-income countries [1, 2]. This chronic neurological condition is the most serious health consequence of the lifecycle of *Taenia solium*, the parasite which causes the disease. Humans acquire NCC by ingesting tapeworm eggs shed in the feces of someone infected with an adult intestinal tapeworm. Once ingested the eggs release oncospheres that penetrate the intestinal wall and disseminate to form cysts throughout the body including the brain. As these cysts degenerate they can provoke an inflammatory process that may produce seizures [3]. A persistent calcified lesion may result and become foci for chronic seizure activity [4,5]. Not all NCC infections are symptomatic and it remains unclear why some people develop seizures while others do not.

Neuroimaging by either computerized tomography (CT) or magnetic resonance imaging (MRI) is required for diagnosis of NCC, yet these tools are often unavailable in areas where *T*. *solium* is endemic. Nonetheless there have been several communities-based studies in Latin-American countries which have evaluated the association between epilepsy or headache and NCC [6–10]. Others studies have evaluated the association between epilepsy or headache and positive serology for cysticercosis [11–12]. Only two studies provide a direct measure of the prevalence of NCC; they both find that a high proportion of asymptomatic or serologically negative persons had NCC [7,10]. We conducted a cross sectional study in northern Peru using CT scan, serology and symptoms survey to provide an estimate of the background prevalence of NCC in endemic areas.

## Methods

### Study site and participants

In 2005, we conducted a cross-sectional study in the rural village of Rica Playa (n = 454), Tumbes, Peru, a region where cysticercosis is endemic. This village was chosen by convenience as it was the site of an ongoing study of porcine cysticercosis. The population in this region is mostly mestizo, a mixture of Spaniard and Amerindian. It is common for residents of this area to raise pigs for consumption and sale by allowing the animals to roam and forage. This practice exposes pigs to human feces, as open defecation is common.

### Study procedures

We conducted a door-to-door survey collecting household level information on the presence of latrine, water source, number of inhabitants and animal husbandry practices. All residents aged 2 years and older were invited to provide a 5ml blood sample to be evaluated for antibodies against *T*. *solium*, and to answer a 14-question neurologic survey to screen for a history of seizures and headaches (appendix); the survey was completed by a parent or legal guardian if the participant was less than 12 years old. This survey was developed in Ecuador and later validated in Peru [11, 13–15]. We offered in-home clinical evaluation by a neurologist to all participants who screened positive for a history of seizures or severe headaches. The International League Against Epilepsy classification of 1993 and the 2^nd^ Edition of The International Classification of Headache Disorders guidelines were applied to diagnose the disorders [16, 17]. All residents aged 18 years and older were also invited to receive a non-contrast computerized tomography (CT scan) of the head to evaluate for lesions consistent with neurocysticercosis. Participants who agreed to receive a CT scan were transported to the clinical facilities at the Center for Global Health Tumbes, where the exam was performed using a helicoid model (Siemens AG, Germany). The CT images were read independently by two radiologists who were blind to the symptoms and serology results of each participant. Women of reproductive age had a urine pregnancy test before receiving the CT scan. There were no exclusion criteria other than age.

Sera were separated using a centrifuge and samples were stored at -20°C until processing at the Instituto de Ciencias Neurológicas, Lima. Samples were analyzed by enzyme-linked immunoelectrotransfer blot for presence of antibodies against *T*. *solium* cysts using lentil-lectin purified glycoprotein antigens (LLGP-EITB) as previously described [18]. The LLGP-EITB assay uses a semi-purified fraction of homogenized *T*. *solium* cysts containing 7 glycoprotein antigens named after the kDa molecular weights of the corresponding reactive bands (GP50, GP42, GP24, GP21, GP18, GP14, GP13). Reactions to any of these 7 glycoprotein antigens are considered positive. This assay is reported to be 100% specific to exposure to T. solium cysticercosis in humans, although it does not distinguish active infection from exposure (see supplemental table) [18]. The sensitivity is reported to be 98% when more than one viable cysts are present, but it is substantially lower for single viable cysts or when only calcified cysts are present [19,20].

We analyzed data using STATA SE12 (StataCorp; College Station, TX). We used two-sided Fisher’s exact test to evaluate differences in proportions. We constructed binomial family general linear models with a logit link to evaluate associations between individual variables and odds of both seropositivity and presence of brain calcifications. Variables significant at the level of *p*<0.25 were retained in the multivariable models in which adjusted odds ratios were estimated. We then used negative binomial family general linear models with log link to estimate rate ratios and 95% confidence intervals for variables associated with the number of brain calcifications. Cluster-robust standard errors were used in all models to account for intrahousehold clustering and model fit was evaluated using Akaike Information Criteria.

Geospatial analysis was conducted in ArcGIS version 10.2.1 with data projected from WGS84 to UTM 17S. 5 households were removed from this analysis because they were outside the town center. We calculated Moran’s I and Getis-Ord General G statistics to evaluate the spatial distribution of calcifications (number of calcifications per household) and seropositivity (number of household residents with ≥3 reactive bands on LLGP-EITB) using squared inverse spatial weighting and 200 meter distance thresholds. P-values were determined using the randomization null hypothesis.

### Ethical considerations

The study protocol and consent forms were reviewed and approved by the institutional review board of the Universidad Peruana Cayetano Heredia. We provided a written informed consent form and a detailed oral explanation to all potential participants. Informed consent was documented by the participant's signature for adults (18 years or older) or by signature of a parent or guardian for children.

## Results

**Fig 1:**
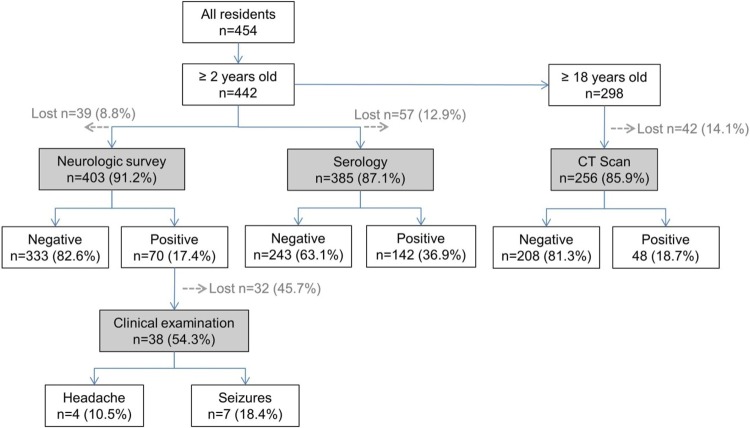
Study flowchart showing eligibility, participation and results of neurologic survey, serology and non-contrast computerized tomography (CT) of the head.

Of the 454 total residents in Rica Playa, 442 were eligible for the study based on age ≥2 years old. Fig 1 and Table 1 shows eligibility, participation and results of the various screening activities. The median age of participants was 28 years (range 2–95 years) and 232 (52.5%) were male. The distribution of participants by serology and head CT results are shown in Table 2. Of the 403 people who participated in the neurologic survey, 43 (10.7%) reported a history of seizures and 33 (8.2%) reported a history of severe headache. Six people (1.5%) reported history of both seizure and headache.

**Table 1:** Number of participants with different result combinations for symptoms, serology and CT scan screening, among the 249 adults that completed all three-study activities.

Screening method			
Symptoms survey	Serology (LLGP-EITB)	CT-scan of head	Number of participants (n = 249)
+	-	-	22
-	+	-	70
-	-	+	17
+	+	-	15
+	-	+	2
-	+	+	23
+	+	+	5
-	-	-	95

**Table 2:** Distribution of individual and household-level characteristics by serologic and radiologic results.

	Serology (LLGP-EITB^†^)		Computerized tomography (non-contrast, head)	
Variable	No. positive (%)	No. negative (%)	No. positive (%)	No. negative (%)
Sex				
Male	75 (39.1)	117 (60.9)	23 (16.4)	117 (83.6)
Female	67 (34.7)	126 (65.3)	25 (21.6)	91 (78.5)
No. residents per household				
1–5	77 (41.4)	109 (58.6)	21 (15.4)	115 (84.6)
6–7	38 (38.0)	62 (62.0)	17 (27.9)	44 (72.1)
8–10	27 (27.3)	72 (72.7)	10 (17.0)	49 (83.1)
Latrine in household				
Yes	69 (36.9)	118 (63.1)	17 (13.7)	107 (86.3)
No	73 (36.9)	125 (63.1)	31 (23.5)	101 (76.5)
Pigs raised at household				
Yes	126 (38.2)	204 (61.8)	41 (18.5)	181 (81.5)
No	16 (29.1)	39 (70.9)	7 (20.6)	27 (79.4)

^†^ Lentil-lectin glycoprotein purified enzyme-linked immunoelectro transfer blot

**Fig 2:**
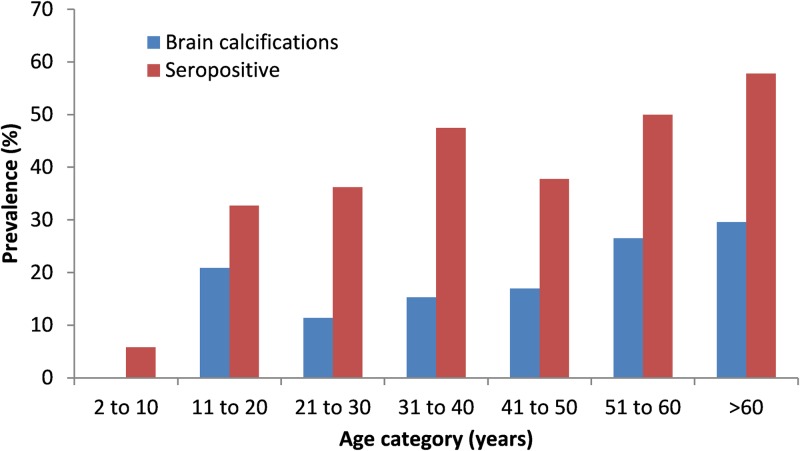
Prevalence of brain calcifications non-contrast computerized tomography (CT) of the head and seroprevalence of antibodies against Taenia solium cysticercosis (LLGP-EITB) by age-group.

Of the 385 individuals who provided a blood sample, 36.9% (n = 142; 95% CI: 32.2 to 41.8) were seropositive for antibodies against cysticercosis. However, the majority (113/142) of seropositive participants denied any history of seizures or severe headache (79.6%; 95% CI: 72.1 to 85.5). Results of bivariable and multivariate analyses are shown in Table 3. After controlling for other variables, age and pig ownership remained statistically associated with seropositivity. For every 1-yr increase in age, there was a 3% increase in the odds of seropositivity (OR 1.03; 95% CI 1.02 to 1.04) (Fig 2). Among adults aged 18 years and older, the seroprevalence was 45.4% (119/262) (95% CI 39.4 to 51.5%). Pig ownership nearly doubled the odds of seropositivity (OR 1.87; 95% CI 1.07 to 3.25).

**Table 3:** Association between individual and household-level characteristics and serologic or evidence of cysticercosis.

	Serology (LLGP-EITB^†^)			
Variable	OR, crude*	*p*	OR, adj.**	95% CI
Age	1.03	<0.01	1.03	1.02–1.04
Female	0.83	0.38	--	--
Owns pigs	1.51	0.09	1.87	1.07–3.25
Latrine present	1.00	1.0	--	--
Number of residents	0.91	0.04	0.96	0.87–1.06
Symptom screening:				
Headache	1.26	0.48	--	--
Seizure	1.53	0.28	--	--

^†^ Lentil-lectin glycoprotein purified enzyme-linked immunoelectro transfer blot

* Bivariable regression using binomial family generalized lineal model with logit link

** Multivariable regression using binomial family generalized lineal model with logit link and retaining variables with *p*<0.25 on bivariable analysis

The prevalence of brain calcifications on CT scan among adults aged 18 years and older was 18.8% (48/256) (95% CI 14.4 to 24.0%). However, the majority of participants with calcification denied any history of seizure or severe headache (40/48, 83.3%; 95% CI 69.5 to 91.7). Of the 48 people with calcifications, 32 (66.7%) had a single calcification, 14 (29.2%) had between 2–10, and 2 (4.2%) had 11 or more. One participant had a single cystic parenchymal lesion in addition to having calcifications. There was no difference in the proportion of participants with calcifications among those who reported headache and/or seizure (8/48, 17.0%) versus those who were asymptomatic (40/209; 19.1%, p = 0.8). Although CT scan and antibody positivity were significantly associated in this population (McNemar χ^2^, p<0.001), this association was far from perfect. Only 28 of 47 CT positive individuals (59.6%) were positive on LLGP-EITB, and 85 out of 202 CT negative individuals (42.1%) were LLGP-EITB positive.

**Fig 3:**
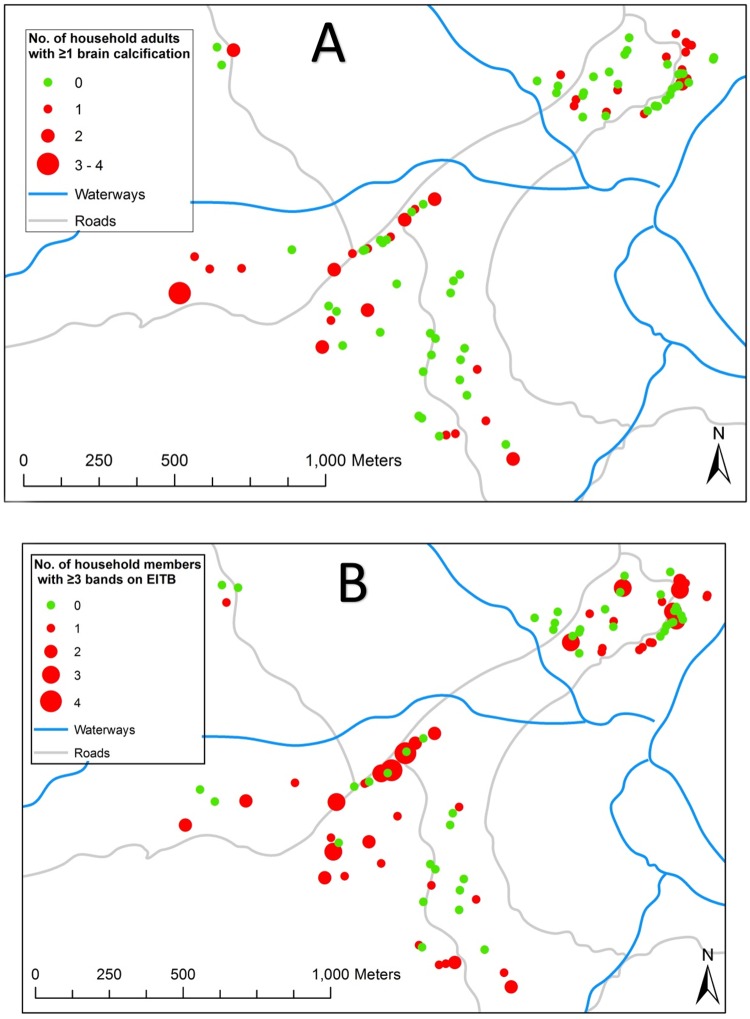
Maps of Rica Playa, Peru, showing location of **A)** the number of brain calcifications per household as detected by non-contrast computerized tomography (CT) of the head, and **B)** the number of household residents seropositive with ≥3 reactive bands on LLGP-EITB. There was no evidence of clustering of either brain calcifications (I = 0.06, p = 0.64; G = 4.6x10-5, p = 0.64) or seropositivity (I = -0.27, p = 0.11; G = 6.8x10-5, p = 0.55) using Moran’s I and Getis-Ord tests respectively.

Age, seropositivity and lack of a household latrine were significantly associated with the presence of brain calcifications after controlling for other factors (Table 4). For every 1-yr increase in age the odds of having brain calcifications increased by 3%. The number of calcifications present also increased by 3% with each 1-yr age increase. Brain calcifications were twice as likely among seropositive people compared to those who were seronegative. However, latrines were protective against having calcifications, with the odds of having brain calcifications reduced in half if there was a latrine at the household. There was no evidence of geospatial clustering of calcifications or seropositivity (Fig 3A and 3B).

**Table 4:** Association between individual and household-level characteristics and evidence of brain calcifications on non-contrast computerized tomography of the head.

	Presence of calcifications				Number of calcifications			
Variable	OR, crude*	*p*	OR, adj.**	95% CI	IRR, crude^§^	*p*	IRR, adj. ^§§^	95% CI
Age	1.02	0.03	1.02	1.00–1.04	1.03	<0.01	1.03	1.01–1.05
Female	1.40	0.33	--	--	2.52	0.02	2.01	1.03–3.93
Owns pigs	0.87	0.77	--	--	0.79	0.70	--	--
Latrine present	0.52	0.04	0.51	0.27–0.96	0.58	0.21	0.57	0.29–1.12
Number of residents	1.07	0.34	--	--	0.93	0.43	--	--
Symptom screening:								
Headache	1.09	0.89	--	--	2.28	0.17	2.47	0.80–7.69
Seizure	1.05	0.92	--	--	0.94	0.88	--	--
Seropositive	2.03	0.02	1.93	1.04–3.58	2.37	0.04	1.81	0.89–3.65

* Bivariable regression using binomial family generalized lineal model with logit link.

** Multivariable regression using binomial family generalized lineal model with logit link. Variables significant at *p*<0.25 on bivariable analysis were retained in this model

§ Bivariable regression using negative binomial family generalized lineal model with log link.

§§ Multivariable regression using negative binomial family generalized lineal model with log link. Variables significant at *p*<0.25 on bivariable analysis were retained in this model.

Participation in follow-up clinical evaluation with the study physician was low which limited our ability to construct models based on clinical diagnosis of headache and seizure. Of the 70 people who reported a history of headache or seizure only 38 (54.2%) agreed to be evaluated by the physician; 25 (65.8%) reported a history of headache, 17 (44.7%) a history of seizure, and 4 (10.5%) a history of both headache and seizure. Of the 38, 4 were confirmed as having epilepsy, 3 as having single non-febrile seizure events, and 4 as having severe headaches. Six of the 7 participants with seizure history provided a blood sample and only one was seropositive; three had CT scan and none had brain calcifications. All 4 of the participants with headache participated in blood sampling and one was seropositive; three underwent CT scan and two had calcifications.

## Discussion

This study found widespread infection with *T*. *solium* in an endemic village in northern Peru where nearly one in five adults had NCC with calcified cysts in the brain. This is one of the only studies of NCC in which neuroimaging was offered in the general population without prior screening, and therefore provides a direct estimate (18.8%) of the prevalence of NCC among adults. Children were not imaged in this study due to the potential risks associated with CT and cumulative radiation.

Most infections were asymptomatic at the time of imaging. Only 17% of those people who had brain calcifications (8/48) reported ever having experienced seizures or severe headaches, the main clinical sequel of NCC. Because calcifications are chronic, it is possible that some of these people will progress to having symptoms in the future. Intermittent peri-lesional edema around calcifications is associated with seizures but the cause and timing of the edema is not well understood [4,5]. However, we cannot evaluate the risk that asymptomatic people with brain calcifications will go on to develop seizures in this cross sectionals study. A longitudinal study is needed to estimate the risk of symptom progression and to understand the factors involved in progression or protection. The fact that in endemic communities most individuals with NCC have asymptomatic calcified brain lesions has been pointed out before [6–10].

All of the NCC cases had calcifications on CT scan; only a single case had a viable cyst. A single calcified lesion was the most common presentation, occurring in two-thirds of the NCC cases. This predominance of single calcifications is typical for NCC in Latin America and has been noted in multiple other studies [6–9,13]. All of these studies, as well as our own, relied on CT scan only, however, which has a lower sensitivity for detecting cysts compared to MRI. It is likely that all of these studies failed to detect some cysts, particularly small parenchymal cysts, and those occurring in the base of the brain or in the extraparenchymal spaces. The only population-based imaging study of NCC to use MRI found cysts in 15.1% of a high-risk, selected asymptomatic population (90/595), although calcifications again predominated (58/90, 64%) [10]. It is not clear how much of this difference is due to imaging modality compared to host-parasite differences.

The 36.9% seroprevalence of antibodies against *T*. *solium* cysticercosis in this study is among the highest reported in Latin America and is consistent with the high prevalence of NCC. Given the rapid rate of seroconversion of the LLGP-EITB from positive to negative, this suggests a highly endemic state with frequent ongoing exposure to *T*. *solium* eggs in the study community [21,22]. The lack of clustering of positive serology or brain calcifications in our study also suggests widespread exposure to the parasite. We noted NCC was twice as likely among people who were seropositive compared to those who were not. Increasing age was the primary risk factor for both seropositivity and NCC, suggesting that continued exposure to the endemic environment increased the risk of acquiring NCC. When compared against CT findings on the population level, the LLGP-EITB has a sensitivity of 59.6% and a specificity of 57.9%. This is consistent with our findings that the main disease presentation in this study community was asymptomatic calcifications and that only a single participant had a live brain cyst. In clinical settings, the sensitivity of the LLGP-EITB for patients with multiple live brain cysts is of 98% and specificity 100%, with stronger antibody responses (4–7 bands) increasing the likelihood of have active NCC lesions. The presence of a latrine was protective against NCC though not against positive serology. At first glance, this may appear paradoxical. However, this could reflect broad low-level exposure to tapeworm eggs within the overall community with risk of infection concentrated in areas with poorest sanitation. There was no evidence, however, of spatial clustering of calcifications or seropositivity within the community.

This study had several limitations in addition to those previously mentioned. This was a single small study in northern Peru in a community with extremely high endemic transmission. The results may not be generalizable to other regions where underlying conditions are different. Although clinical evaluation and verification of symptoms was planned, nearly half of those who reported symptoms refused evaluation by the neurologist for religious reasons despite our efforts to obtain support from local church leaders. We therefore rely on self-report of symptoms in the analysis, for which the positive predictive value is known to be low.

In conclusion, this study reports a high prevalence of NCC infection in an endemic community in Peru and confirms that a large proportion of apparently asymptomatic residents have brain calcifications that could provoke seizures in the future. Long-term follow-up of these individuals could provide an estimate of the risk of symptom development. Effective control interventions are needed in *T*. *solium* endemic regions around the world to reduce the incidence of disease.

## Supporting Information

S1 Checklist Strobe (PDF)

S1 Neurological survey 14q (PDF)
