# Supplementary material for: High Prevalence of Asymptomatic Neurocysticercosis in an Endemic Rural Community in Peru
Source: PLoS Negl Trop Dis. 2016 Dec 19;10(12):e0005130. doi: 10.1371/journal.pntd.0005130 (PMC5167259; doi:10.1371/journal.pntd.0005130)
Supplement: S1 Neurological survey 14q — (PDF) [file pntd.0005130.s002.pdf]

P1
¿Alguna vez ha tenido movimientos en los brazos o piernas que no pudo controlar?
Si = 1
No = 0

P1 a
Indica si más de una vez el paciente ha tenido los síntomas de la pregunta anterior
Si = 1
No = 0

P2
¿Alguna vez ha tenido ataques en los que se cae y se pone pálido?
Si = 1
No = 0

P2 a
Indica si más de una vez el paciente ha tenido los síntomas de la pregunta anterior
Si = 1
No = 0

P3
¿Alguna vez ha tenido ataque en los que se cayó y perdió el conocimiento?
Si = 1
No = 0

P3 a
Indica si más de una vez el paciente ha tenido los síntomas de la pregunta anterior
Si = 1
No = 0

P4
¿Alguna vez ha perdido el conocimiento?
Si = 1
No = 0

P4 a
Indica si más de una vez el paciente ha tenido los síntomas de la pregunta anterior
Si = 1
No = 0

P5
¿Alguna vez ha tenido ataques en los que se cayó y se mordió la lengua?
Si = 1
No = 0

P5_a
Indica si más de una vez el paciente ha tenido los síntomas de la pregunta anterior
Si = 1
No = 0

P6
¿Alguna vez ha tenido ataques en los que se cayó y se orinó?
Si = 1
No = 0

P6_a
Indica si más de una vez el paciente ha tenido los síntomas de la pregunta anterior
Si = 1
No = 0

P7
¿Alguna vez ha tenido ataques en los que pierde el contacto con las cosas que lo rodean y hu[illegible]
Si = 1
No = 0

P7_a
Indica si más de una vez el paciente ha tenido los síntomas de la pregunta anterior
Si = 1
No = 0

P8
¿Alguna vez ha tenido ataques en los que ve luces brillantes o de colores?
Si = 1
No = 0

P8_a
Indica si más de una vez el paciente ha tenido los síntomas de la pregunta anterior
Si = 1
No = 0

P9
¿Alguna vez alguien le ha dicho que sufre de ataques de epilepsia?
Si = 1
No = 0

P9_a
Indica si más de una vez el paciente ha tenido los síntomas de la pregunta anterior
Si = 1
No = 0

P10
¿Ha tenido algún síntoma como los de arriba?
Si = 1
No = 0

P10_ma
Indica el mes y el año en el que tuvo los síntomas de P10
mm/aaaa

P_10a
Indica desde cuando tiene los síntomas de la pregunta anterior

1. Menos de un año
2. Entre uno y dos años
3. Entre dos y tres años
4. Entre tres y cinco años
5. Mas de cinco años

P11
¿Sufre de dolores de cabeza?
Si = 1
No = 0

P11_a
Cuando está con dolor de cabeza

1. Interrumpe lo que esta haciendo
2. Hace mal lo que esta haciendo
3. No le da importancia

P11_b
Como se alivia?

1. Sin medicamento
2. Con medicamento

P11_b1
¿Qué medicamentos utiliza para aliviarse?

1. Natural
2. Pastillas
3. Inyecciones

P11_c
¿Desde cuando le duele la cabeza?

1. Menos de un año
2. Entre uno y dos años
3. Entre dos y tres años

4. Entre tres y cinco años
5. Mas de cinco años

P11_ma
Indica el mes y año en el que tuvo los síntomas de P11_c
mm/aaaa

P12
¿Ha tenido cambios en su carácter?
Si = 1
No = 0

P12_ma
Indica el mes y año en el que tuvo los síntomas de P12
mm/aaaa

P12_a
¿Desde cuando tiene los síntomas?
1. Menos de un año
2. Entre uno y dos años
3. Entre dos y tres años
4. Entre tres y cinco años
5. Mas de cinco años

P13
¿Ha notado cambios en su conducta o manera de actuar?
Si = 1
No = 0

P13_ma
Indica el mes y año en el que tuvo los síntomas de P13
mm/aaaa

P13_a
¿Desde cuando tiene los síntomas?
1. Menos de un año
2. Entre uno y dos años
3. Entre dos y tres años
4. Entre tres y cinco años
5. Mas de cinco años

P14
¿Alguna vez se le ha paralizado alguna parte del cuerpo?
Si = 1
No = 0

P14 a

¿Qué duración tuvo el síntoma de la pregunta anterior?

1. Un día
2. Dos a tres días
3. tres días a tres semanas

Mas de tres semanas
